# Potential role for the tumor suppressor CYLD in brain and notochord development

**DOI:** 10.1111/1759-7714.13973

**Published:** 2021-05-13

**Authors:** Te Li, Yiyan Wang, Dengwen Li, Jun Zhou, Bo Zhang, Xianfei He

**Affiliations:** ^1^ State Key Laboratory of Medicinal Chemical Biology, College of Life Sciences Nankai University Tianjin China; ^2^ Key Laboratory of Cell Proliferation and Differentiation of the Ministry of Education, Peking University Genome Editing Research Center, College of Life Sciences Peking University Beijing China; ^3^ Shandong Provincial Key Laboratory of Animal Resistance Biology, Collaborative Innovation Center of Cell Biology in Universities of Shandong, Institute of Biomedical Sciences, College of Life Sciences Shandong Normal University Jinan China

**Keywords:** cylindromatosis, tumor suppressor, CYLD, TALEN, brain

## Abstract

**Background:**

The cylindromatosis (CYLD) tumor suppressor is a microtubule‐associated deubiquitinase that plays a critical role in the regulation of cell signaling and contributes to a variety of physiological and pathological processes. However, the functions of CYLD in zebrafish are less well known, particularly with regard to their development and physiology. In this context, we investigated the loss of function of CYLD in zebrafish via transcription activator‐like effector nuclease (TALEN)‐based gene deletion.

**Methods:**

Semi‐quantitative RT‐PCR was used to quantify CYLD mRNA expression in zebrafish embryos at various developmental stages. We also performed whole‐mount in situ hybridization to further assess the dynamic expression and distribution of CYLD in the entire zebrafish embryos at different stages. In addition, we deleted CYLD in zebrafish with TALENs to investigate its potential impact on embryonic development.

**Results:**

The expression of CYLD mRNA varied during early embryonic development. The CYLD mRNA localized to the brain and notochord of developing zebrafish embryos. Homozygous deletion of CYLD resulted in embryonic death before 8 h post‐fertilization.

**Conclusions:**

CYLD appears to play an important role in central nervous system development in zebrafish. Although severe embryonic death restricted analysis of homozygous mutants, further research into the role of CYLD in central nervous system development is warranted.

## Graphical abstract

Cylindromatosis (CYLD) has important pathophysiological roles in humans. More than just a tumor suppressor, CYLD has many potential functions. We exploited the zebrafish model to investigate dynamic changes in CYLD expression during embryonic development. In doing so, we discovered a potential role for CYLD in brain and notochord development, providing experimental evidence for a novel pathophysiological role in central nervous system development.

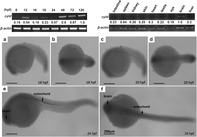

## INTRODUCTION

The cylindromatosis (CYLD) tumor suppressor is a deubiquitinating enzyme that mainly removes K63‐linked polyubiquitin chains from a variety of signal transduction‐related substrates.^1^ CYLD was first identified through a genetic screen of familial cylindromatosis patients.^2^, ^3^ Genome sequencing efforts have revealed somatic CYLD mutations in several human cancers,^4^, ^5^, ^6^, ^7^, ^8^ in which it acts as a tumor suppressor through its effects on cellular proliferation,^9^, ^10^ apoptosis,^11^ migration,^12^ and signal transduction.^13^, ^14^ There are many kinds of deubiquitinating enzymes in mammalian cell, which play their respective functions.^15^, ^16^, ^17^ The CYLD gene encodes a 956 amino acid protein with several functional domains: three N‐terminal CAP‐Gly domains and a C‐terminal ubiquitin‐specific catalytic domain.^18^ The first two CAP‐Gly domains mediate its binding to tubulin and/or microtubules, thereby tightly regulating microtubule stability for cell migration.^19^, ^20^ In addition, CYLD maintains epithelial morphogenesis and homeostasis by regulating mitotic spindle behavior^21^, ^22^ and cell junction assembly^23^, ^24^, ^25^ The last CAP‐Gly domain binds directly to NF‐κB essential modulator (NEMO).^26^

Genetically engineered CYLD mouse models have firmly established a tumor suppressor function for CYLD. Homozygous knockout of CYLD rendered mice more susceptible to papilloma development than wild‐type (WT) littermates following treatment with the chemical carcinogens dimethylbenz[a]anthracene (DMBA) and 12‐O‐tetradecanoylphorbol‐13‐acetate (TPA).^27^ In another study, CYLD knockout mice developed chronic colonic inflammation and colon tumors following treatment with dextran sulfate sodium.^28^ Furthermore, abnormal expression of or mutations in CYLD have been identified in several diseases including lung fibrosis,^29^ osteolytic lesions,^30^ and cardiovascular disease.^31^ Depletion of or loss‐of‐function mutations in CYLD result in excessive increase of intracellular ubiquitination level. Undoubtedly, excessive ubiquitination overly promotes activation of TGF‐β, Wnt, and mTOR signalings. The molecular mechanism of these diseases can be attributed to the aberrant signalings. These findings indicate that CYLD has important pathophysiological roles in humans.

However, few studies have investigated the role of CYLD in organ development. Embryonic development is a rapid, dynamic, and complex process. While mice provide a useful model of embryonic development, zebrafish (*Danio rerio*) provide a rapid, visible, and easily manipulable model of organ development. We therefore exploited the zebrafish model to investigate dynamic changes in CYLD expression during embryonic development. In doing so, we discovered a potential role for CYLD in brain and notochord development, providing experimental evidence for a novel pathophysiological role in central nervous system development.

## METHODS

### Zebrafish husbandry

Zebrafish were kept at 28.5°C, pH 7.2–7.6, in an animal holding facility (14 h light/10 h dark photoperiod). Hatched fry were fed paramecia from five to 14 days post‐fertilization (dpf), after which juvenile fish were fed live brine shrimp larvae. All zebrafish experiments were conducted according to the guidelines and approval of the Institutional Animal Research and Ethics Committees.

### RNA isolation and RT‐PCR

At each time point, 15 zebrafish embryos were sacrificed and total RNA was extracted using TRIzol reagent (Invitrogen, 15 596 026) followed by determination of RNA concentration and purity using the NanoDrop Lite UV spectrophotometer (Thermo Fisher Scientific). cDNA was synthesized by reverse transcription with the PrimeScript RT Reagent kit (Takara Bio, RR047A), and semi‐quantitative RT‐PCR was conducted with the Takara Ex Taq (Takara, RR001A) on an ABI Veriti System. The primers for the main zebrafish homolog of human CYLD were as follows: CYLD forward 5′‐ATCATTCAGATGCCTCGGTTTGG‐3′ and reverse 5′‐AACAGCTCCATCTGCTGGTGAGG‐3′; and for β‐actin (actb1; housekeeping control): forward 5′‐TCCCCTTGTTCACAATAACCTAC‐3′ and reverse 5′‐GGTCACAATACCGTGCTCAAT‐3′.

### Whole‐mount in situ hybridization

For whole‐mount in situ hybridization, the cDNA for the probe was cloned into pESAY‐T vectors. Antisense RNA probes were synthesized using T7 RNA polymerase (Roche Applied Science, 10881767001) and DIG RNA labeling mix (Roche Applied Science, 11277073910). In situ hybridization was performed according to a standard protocol in our laboratory.

### Protein sequence alignment

Zebrafish (*Danio rerio*), mouse (*Mus musculus*), and human (*Homo sapiens*) genomic sequences were obtained from the Ensembl Genome Browser (http://www.ensembl.org/). Clustal Omega (https://www.ebi.ac.uk/Tools/msa/clustalo/) was used for sequence alignment and comparisons with 5‐aa windows and a 50% identity threshold.

### Targeted gene disruption of CYLD by TALENs

pCS2‐TALEN‐pedas/perr vectors were constructed as described.^32^ TALENs assembly and microinjection into one‐cell stage zebrafish embryos were conducted according to previous studies.^32^ Embryos injected with TALENs were allowed to grow to adulthood and then crossed with wild‐type (WT) zebrafish. Eight F1 embryos were collected from each pair of F0 fish, and genomic DNA was extracted for genotyping. F1 zebrafish embryos were then raised to 3 dpf, screened for heterozygosity, and then self‐crossed to generate the F2 generation. Zebrafish tail genomic DNA was extracted using TRIzol reagent (Thermo Fisher Scientific, 15 596 026) according to the manufacturer's instructions. The primers were as follows: cyldu, forward 5′‐AGTAGGAGAGCACGTTTCTCTCCTGTT‐3′ and reverse 5′‐GGTTTCAGCCGTCCGCCTT‐3′; cyldd, forward 5′‐AATAGGTCATGTTATAAAGAGCTGATTTCC‐3′ and reverse 5′‐ACAGAGATAATAAAGTCCACCTGGCTTC‐3′.

### Statistical analysis

All experiments were performed three times independently. Quantitative data are expressed as means ± SEM. Statistical significance was determined using a two‐tailed Student's *t*‐test. A *p*‐value<0.05 was considered statistically significant.

## RESULTS

### Changes in CYLDA expression during early development

**FIGURE 1:**
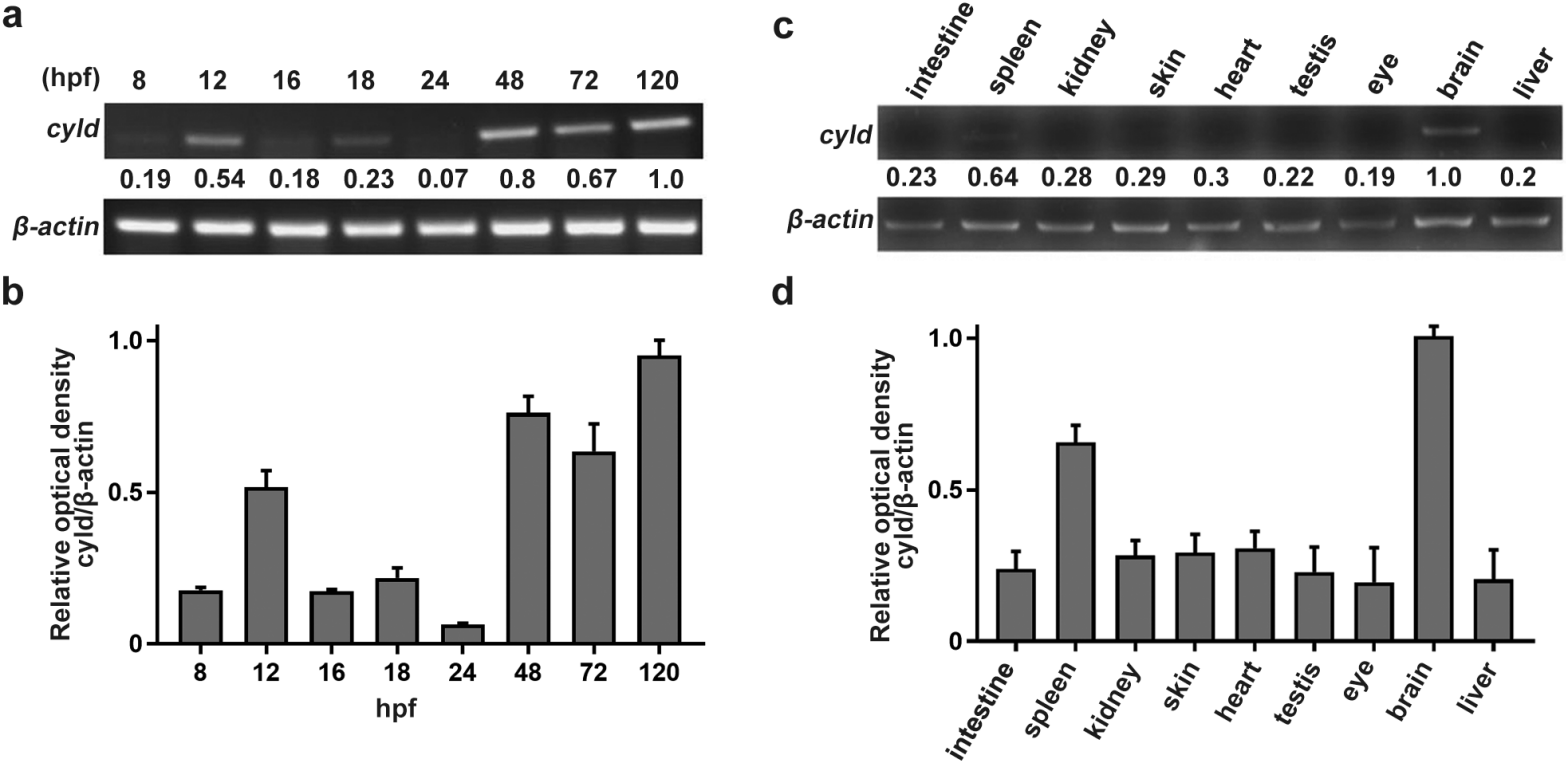
CYLD expression varies across the time course of early development and is localized to the brain. (a) CYLD expression at different stages of zebrafish embryogenesis. (b) Quantification of CYLD expression in zebrafish embryos (mean ± SEM from three experiments). (c) CYLD expression in the primary organs of zebrafish. (d) Quantification of CYLD expression in zebrafish organs (mean ± SEM from 3 experiments)

Unlike in humans, there are two CYLD paralogs in zebrafish: CYLDA and CYLDB, with CYLDA conserved in mammals. We therefore studied CYLD (CYLDA) expression by RT‐PCR in whole embryos at several representative developmental stages of zebrafish. CYLD expression fluctuated dramatically during the early stages of development. Before 12 h post‐fertilization (hpf), CYLD was expressed at relatively low levels. Unexpectedly, however, CYLD expression increased significantly at 12 hpf before decreasing gradually until 24 hpf and increasing again to high levels after 48 hpf (Figure 1(a) and (b)).

### CYLD is principally expressed in the brain and notochord

**FIGURE 2:**
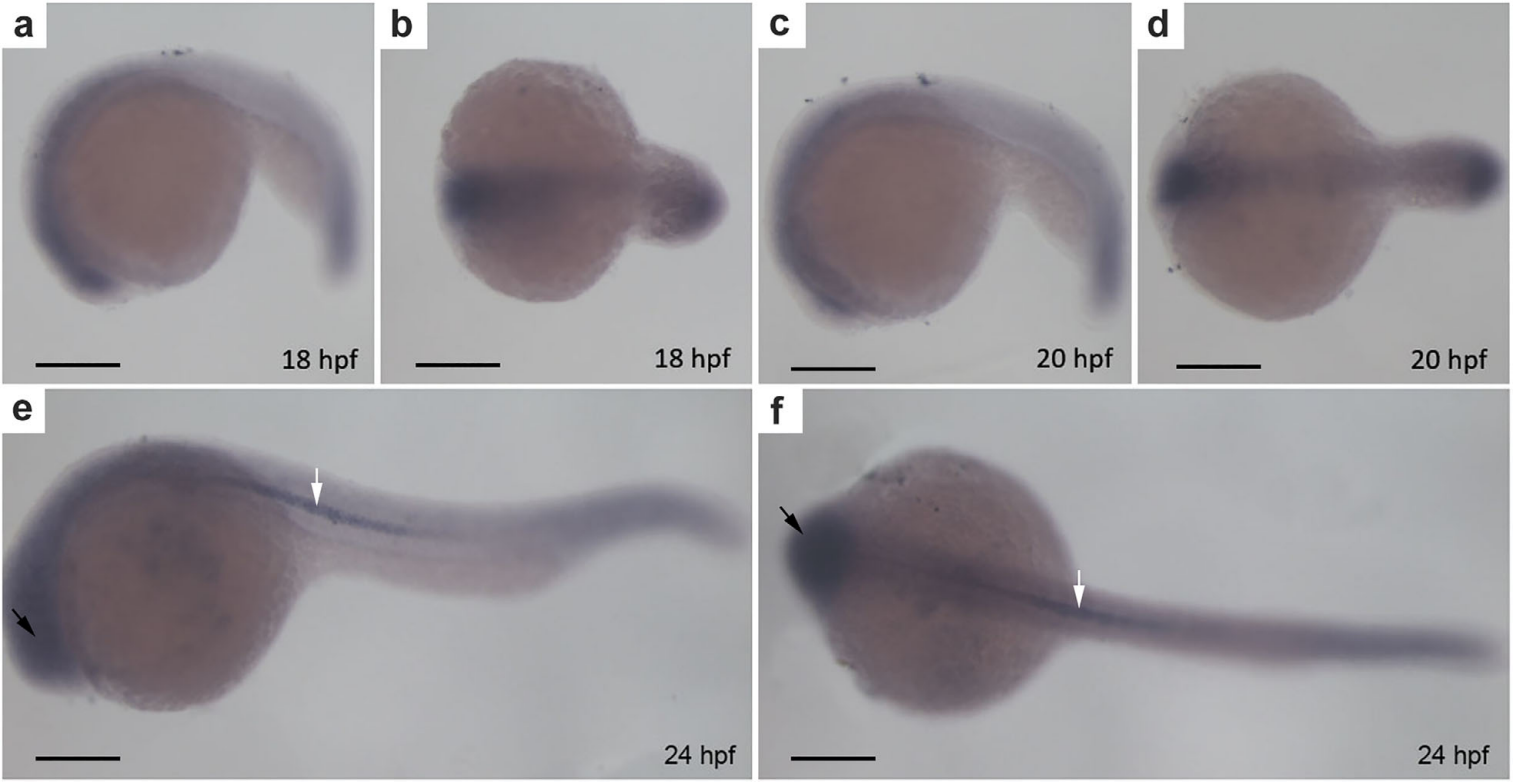
CYLD mainly localizes to the brain and notochord. (a–f) Whole‐mount in situ hybridization of zebrafish embryos. (a–b), (c–d), (e–f) side and top views of zebrafish embryos at 18 hpf, 20 hpf, and 24 hpf, respectively. The black arrows represent the brain of the zebrafish. The white arrows represent the notochord of the zebrafish. Scale bar = 250 μm

We next examined the expression of CYLD in different organs. CYLD was mainly expressed in the brains and spleens of adult zebrafish (Figure 1(c) and (d)). To determine the precise localization of CYLD in the embryo, whole‐mount in situ hybridization was performed in 18, 20, and 24 hpf zebrafish siblings. CYLD was primarily expressed in the brain and notochord in zebrafish embryos (Figure 2(a)–(f)).

### CYLD is highly conserved in humans, mice, and zebrafish

**FIGURE 3:**
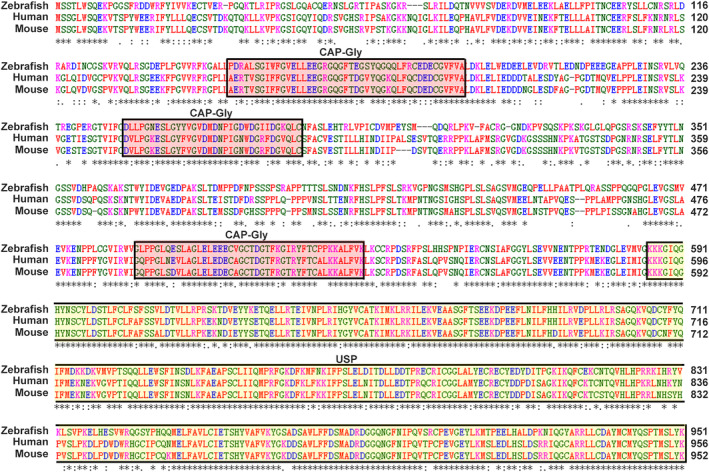
CYLD is highly conserved. Sequence alignment of CYLD in humans, mice, and zebrafish. ClustalW was used for alignment. Asterisks indicate positions that have a single, fully conserved residue; colons and stops indicate that the stronger‐ and weaker‐score groups are fully conserved, respectively. The main domains are represented in the box

As previously reported, CYLD is a highly conserved and vital deubiquitinating enzyme in mammals. To analyze the conservation of CYLD in zebrafish, the ClustalW algorithm was used to align human, mouse, and zebrafish sequences. There was up to 69% identity of CYLD in zebrafish, humans, and mice. Several conserved domains were apparent in the alignment, namely three CAP‐Glys and a C‐terminal hydrolase domain (Figure 3).

### CYLD deleted zebrafish mutants by TALENs

**FIGURE 4:**
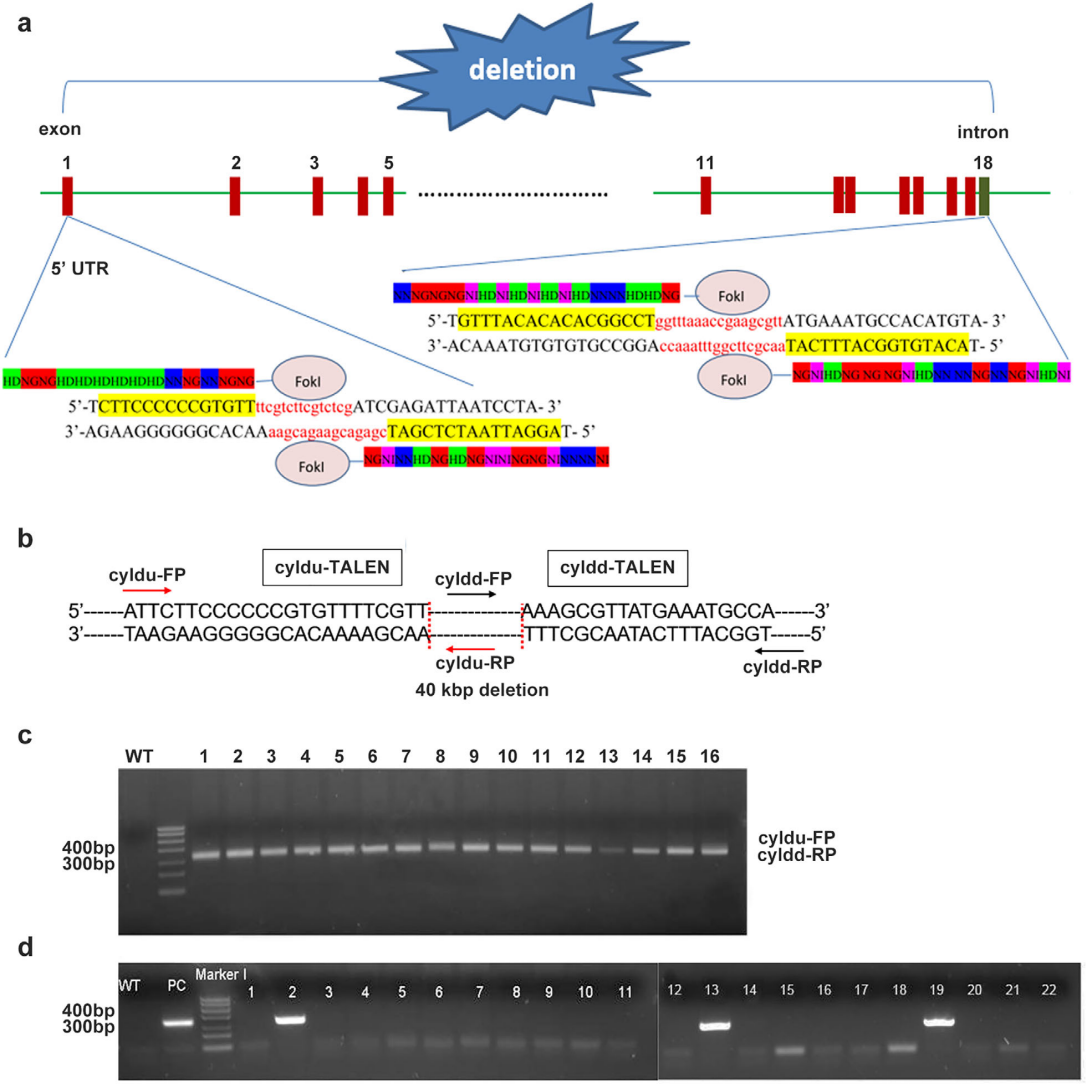
Construction of CYLD‐deleted zebrafish by TALENs. (a) Schematic of the genomic locations of the two CYLD ‐TALEN targets. (b) the schematic of the PCR primers for genotyping. (c) Evaluation of large‐fragment deletion efficiency of *cyld* mediated by TALENs. (d) CYLD F0 mutant screening by genotyping

To interrogate the function of CYLD in brain and notochord development, TALENs were used to construct CYLD‐deleted mutants. Two TALENs targets were selected, the middle of the first exon and the end of the eighteenth exon, respectively (Figure 4(a)). For genotyping, two primer sets were designed, cyldu‐FP/RP and cyldd‐FP/RP (Figure 4(b)). First, 16 groups (five eggs each) of fertilized zebrafish eggs were injected with equal proportions of TALENs mRNA. Compared to untreated embryos, a ~350 bp band was observed by PCR with primer set cyldu‐FP/cyldd‐RP after injection (Figure 4(b)). Therefore, the TALENs effectively cleaved the CYLD locus. The 350 bp PCR product was sequenced to confirm that the target 40‐kb fragment was removed from CYLD. In total, we obtained 22 TALEN‐induced mutants (founders).

### Screening of germline F0 and F1 mutants

**FIGURE 5:**
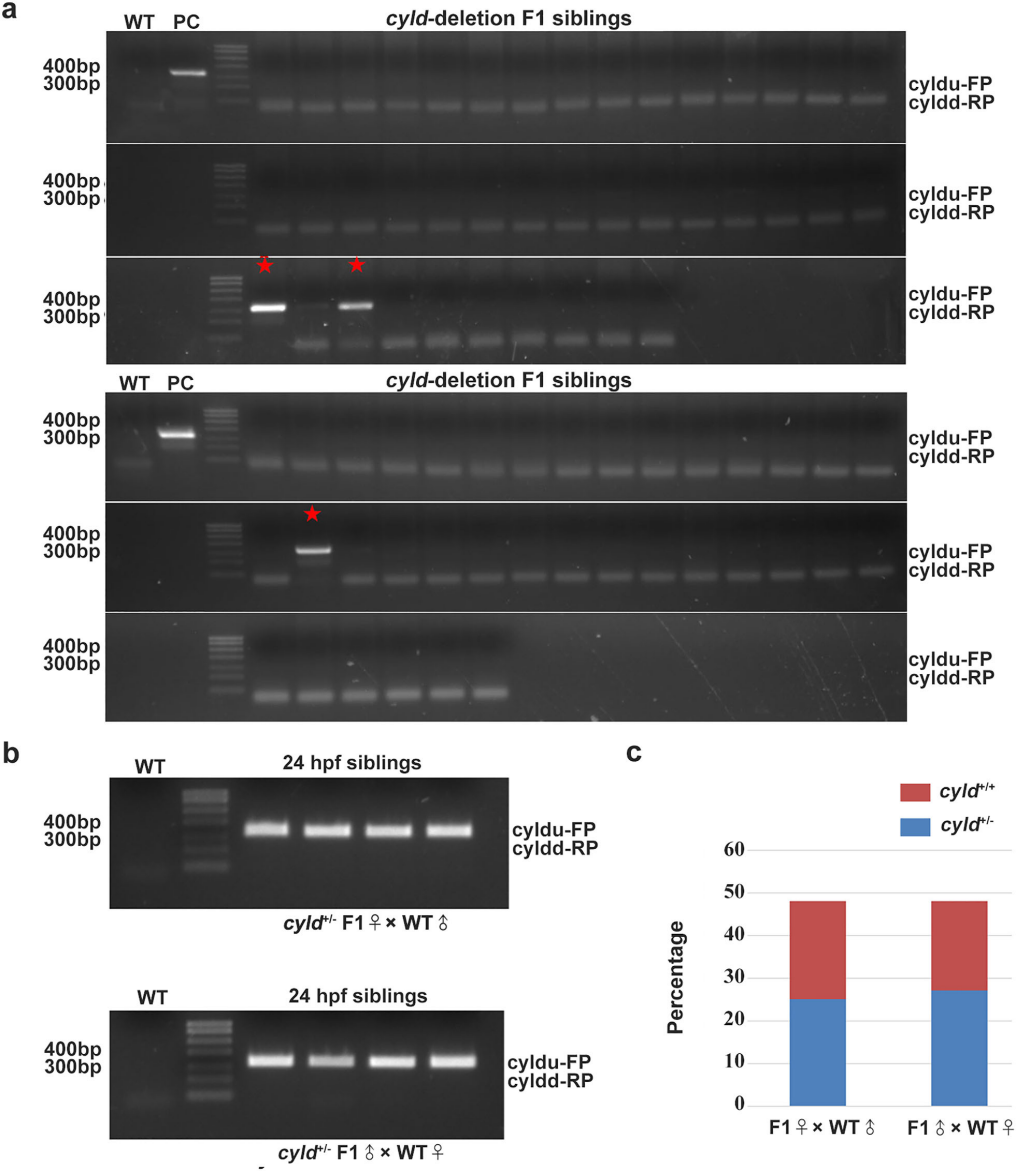
Heterozygous mutants were reproducible. (a) CYLD F1 mutant screening by genotyping. The red asterisk indicates heterozygous mutants. (b–c) Analysis of the female and male gametes of the CYLD F1 mutants

To obtain reproductive CYLD‐deleted zebrafish, the 22 founders were crossed with wild‐type zebrafish. After genotyping, three positive founders were identified and retained (Figure 4(d)). Next, F0 were bred with wild‐type zebrafish to obtain F1. We found that CYLD^+/−^ individuals were ~3.75% (Figure 5(a)). F1 males or females were crossed with wild‐type zebrafish, and the genotypes of all 24 hpf siblings were characterized by PCR. The number of CYLD^+/−^ and CYLD^+/+^ individuals was close to 1:1 (Figure 5(b) and (c)).

### Homozygous deletion of CYLD in zebrafish might cause fetal death

**FIGURE 6:**
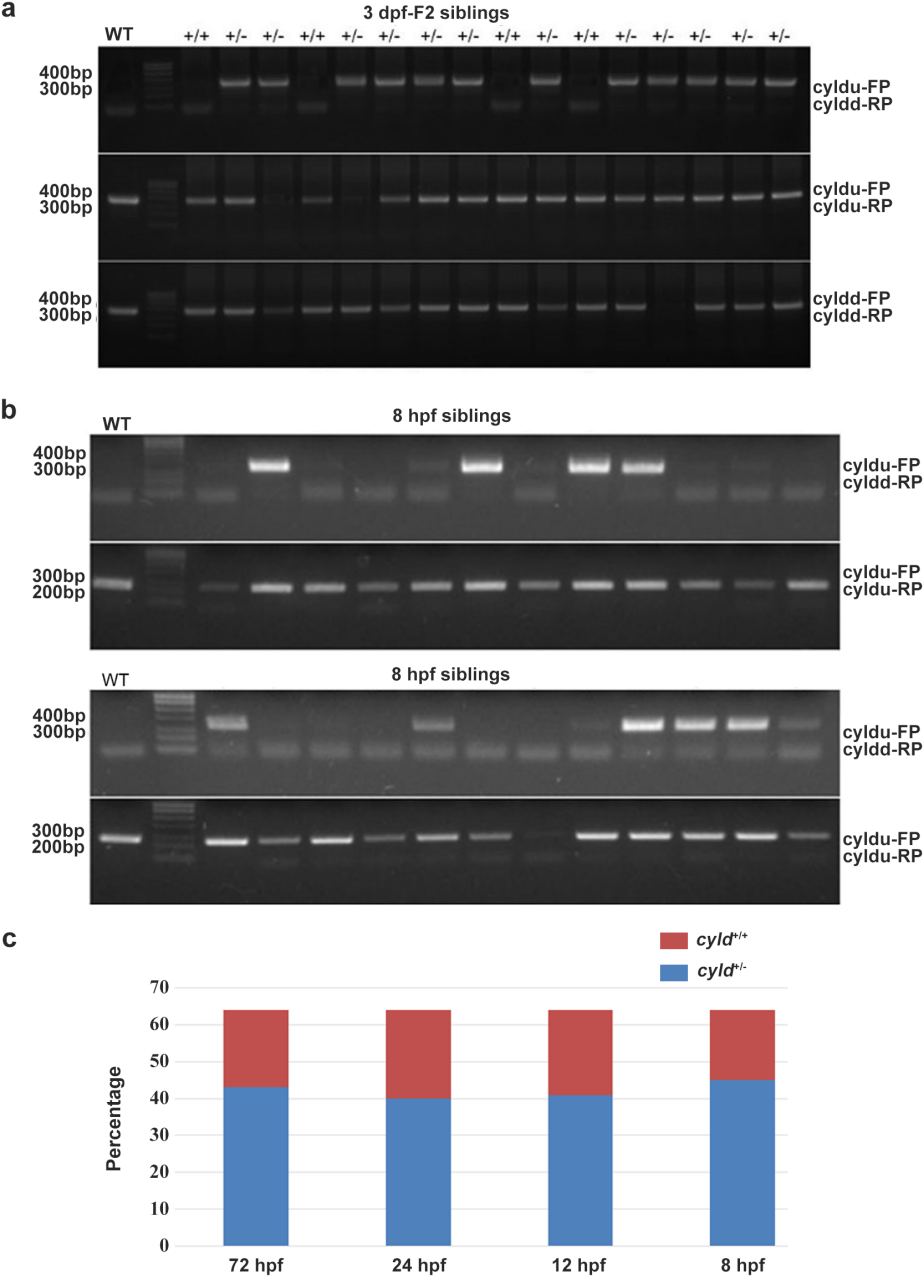
Homozygous deletion of CYLD results in fetal death. (a) 3 dpf of CYLD F2 mutant screening by genotyping. (b) 8 hpf of CYLD F2 mutant screening by genotyping. (c) Statistical results of 8 hpf, 12 hpf, 24 hpf, and 3 dpf mutants with different genotypes, respectively

Since CYLD^+/−^ zebrafish displayed no discernable difference to wild‐type zebrafish, male and female CYLD^+/−^ F1 fish were crossed to produce homozygous F2 zebrafish. In order to distinguish homozygous zebrafish from heterozygous zebrafish, three primer sets (cyldu‐FP/cyldd‐RP, cyldu‐FP/RP and cyldd‐FP/RP) were used. Briefly, for heterozygous zebrafish, three bands were observed with three primer sets. The homozygous zebrafish had only one 350‐bp band with cyldu‐FP/cyldd‐RP. For wild‐type zebrafish, two bands with cyldu‐FP/RP and cyldd‐FP/RP were observed. When analyzed at 3 dpf, no homozygous zebrafish were identified (Figure 6(a)), and CYLD^+/+^ and CYLD^+/−^ offspring were present at a ratio close to 1:2 (Figure 6(c)), consistent with an overall CYLD^*+/+*^: CYLD^+/−:^ CYLD^−/−^ ratio close to 1:2:1 with assumed nonviability of CYLD^−/−^ embryos.

### Embryos die earlier than 8 hpf

To understand the cause of CYLD^−/−^ lethality, the genotypes of sufficient numbers of embryos at different post‐fertilization timepoints were assessed. At all developmental stages, the CYLD^*+/+*^: CYLD^+/−^ ratio in F2 progeny was close to 1:2 (Figure 6(c)), and homozygous knockout zebrafish were not detectable at 8 hpf (Figure 6(b)). Therefore, embryonic death of CYLD^−/−^ embryos was likely to have been earlier than 8 hpf. For unknown reasons, our genotyping method did not work on embryos before 8 hpf.

## DISCUSSION

Compared to mice, zebrafish grow quickly and have transparent bodies, allowing the dynamic study of developmental genes. Before gene editing became widespread, N‐ethyl‐N‐nitrosourea (ENU)‐induced point mutations were an effective way to undertake large‐scale gene function studies.^33^ The mutation efficiency of ENU is up to 0.2%, about 10 times greater than other mutation methods. Moreover, mutations are random and unbiased, making the technique suitable for saturated mutagenesis analysis of the functional zebrafish genome.^34^ Although many mutants can be easily obtained in ENU mutagenesis screening, it is difficult to identify the affected genes in these mutants. With the advent of gene editing, zebrafish have become even easier to study.

Unlike in humans and mice, the zebrafish genome harbors two versions of CYLD, CYLDA and CYLDB. CYLDA is the main zebrafish homolog of human CYLD. We therefore assessed *CYLD* expression in zebrafish and found that CYLD expression levels changed dramatically during early development. Furthermore, CYLD was mainly distributed in the brain and notochord. We hypothesized that CYLD may play an important role in central nervous system development, and constructed a CYLD knockout zebrafish by TALENs. To investigate whether CYLD deletion might inactivate gametes, we crossed female or male heterozygotes separately with wild‐type fish. Interestingly, the ratios of newly hatched CYLD^+/+^ and CYLD^+/−^ zebrafish were almost exactly 1:1, suggesting that CYLD deletion did not affect gamete fertility.

Heterozygous mutants were not visibly different to wild‐type zebrafish. Therefore, the expression of one copy of CYLD seems to be sufficient to maintain normal cellular processes. We continued to reproduce the fish to obtain homozygous knockout individuals by hybridizing heterozygotes. During breeding, many heterozygous and wild‐type zebrafish were noted and, in theory, the CYLD^+/+^:CYLD^+/−^:CYLD^−/−^ ratio should have been close to 1:2:1 rather than the 1:2:0 ratio observed in our breeding experiments. We presumed that CYLD homozygosity resulted in embryonic death. We counted the proportions of the three genotypes at different several stages of development.

Unsurprisingly, no homozygous knockouts were found, even in 8 hpf embryos. In our hands, due to the limitations of PCR, we could not establish whether embryos died earlier than 8 hpf. In mouse studies, complete CYLD knockout was not fatal. This suggests that mammals may be equipped with compensatory pathways and that functional CYLD is essential in zebrafish. This premature death caused by whole body knockout of CYLD currently limits the research, so organ‐specific knockout of CYLD in zebrafish will be used in future studies.

Here, we found that CYLD might play an important role in zebrafish central nervous development. Brain development involves the growth of nerve cells and the formation of synapses.^35^ We assumed that CYLD plays a critical role in brain development. On the one hand, CYLD directly regulates microtubule stability through its CAP‐Glys domain. On the other hand, CYLD may directly regulate brain development through its deubiquitination enzyme function. A recent study found that CYLD variants in frontotemporal dementia were associated with severe memory impairment in a Portuguese cohort.^36^ Although zebrafish are quite different to mammals, they have many advantages as experimental models including transparency and fast growth. Since highly conserved genes have similar functions in all species, we concluded that zebrafish might be useful to establish the important functions of CYLD. Although this work was limited by severe embryonic death, our data show that CYLD may play a role in development, particularly of the central nervous system. Future studies should be performed to clarify its functions in nervous system development.

## CONFLICT OF INTEREST

The authors declared no conflicts of interest.
